# Neuronal Proteomic Analysis of the Ubiquitinated Substrates of the Disease-Linked E3 Ligases Parkin and Ube3a

**DOI:** 10.1155/2018/3180413

**Published:** 2018-03-06

**Authors:** Aitor Martinez, Juanma Ramirez, Nerea Osinalde, Jesus M. Arizmendi, Ugo Mayor

**Affiliations:** ^1^Department of Biochemistry and Molecular Biology, University of the Basque Country (UPV-EHU), 48940 Leioa, Spain; ^2^Department of Biochemistry and Molecular Biology, University of the Basque Country (UPV-EHU), 01006 Vitoria-Gasteiz, Spain; ^3^Ikerbasque, Basque Foundation for Science, Bilbao, Spain

## Abstract

Both Parkin and UBE3A are E3 ubiquitin ligases whose mutations result in severe brain dysfunction. Several of their substrates have been identified using cell culture models in combination with proteasome inhibitors, but not in more physiological settings. We recently developed the ^bio^Ub strategy to isolate ubiquitinated proteins in flies and have now identified by mass spectrometry analysis the neuronal proteins differentially ubiquitinated by those ligases. This is an example of how flies can be used to provide biological material in order to reveal steady state substrates of disease causing genes. Collectively our results provide new leads to the possible physiological functions of the activity of those two disease causing E3 ligases. Particularly, in the case of Parkin the novelty of our data originates from the experimental setup, which is not overtly biased by acute mitochondrial depolarisation. In the case of UBE3A, it is the first time that a nonbiased screen for its neuronal substrates has been reported.

## 1. Introduction

Both Parkin (PARK2) and UBE3A are E3 ubiquitin ligases for which mutations result in severe brain dysfunction, Familial Parkinson's Disease (PD), and Angelman Syndrome (AS). In order to unravel the molecular mechanisms leading to these neurological dysfunctions it is necessary to identify and understand the role of their ubiquitinated substrates. Several substrates of UBE3A and Parkin have been surveyed mostly using cell culture overexpression models in combination with proteasome inhibitors. But more recently, a more physiological setting has been achieved by using an* in vivo* biotinylation strategy to isolate ubiquitinated proteins from* Drosophila *brains. With a label-free mass spectrometry approach, in order to quantify ubiquitinated proteins, we detected substrates of these two E3 ligases in* Drosophila*. This is an example of how flies can be used to reveal physiological substrates of disease-associated proteins. The results, using* Drosophila* as a validated model for neuronal disorders, provide new leads towards the cellular roles of these two disease causing E3 ligases.

## 2. Intracellular Proteostatic Quality Control Mechanisms: The Ubiquitin-Proteasome System (UPS) and Autophagy

The human genome contains ~20.000 protein-coding genes [1], but the set of proteins (proteome) present in a given cell is specifically determined in a cell type and developmental manner [2, 3]. Currently, the deepest proteomic coverage has identified about 12,000 proteins in mice brain samples [4]. In order to adapt their proteomes according to cellular requirements and warrant appropriate fitness of proteins, cells differentially express and regulate their genome through interconnected pathways of protein synthesis and distinct quality control mechanisms [5]. A plethora of cofactors and chaperones supports newly synthesised proteins to ensure their correct folding into fully functional three-dimensional structures [5]. This is a critical process not only to maintain physiological proteostasis but also to avoid the appearance of toxic protein aggregates [6]. However, even when proteins are correctly folded and functionally active in their final compartment, various factors can destabilise the proteins and irreversibly impair them. For this purpose, cells possess quality control mechanisms such as the Ubiquitin-Proteasome System (UPS) and autophagy that specifically degrade damaged proteins and organelles [7, 8].

**Figure 1:**
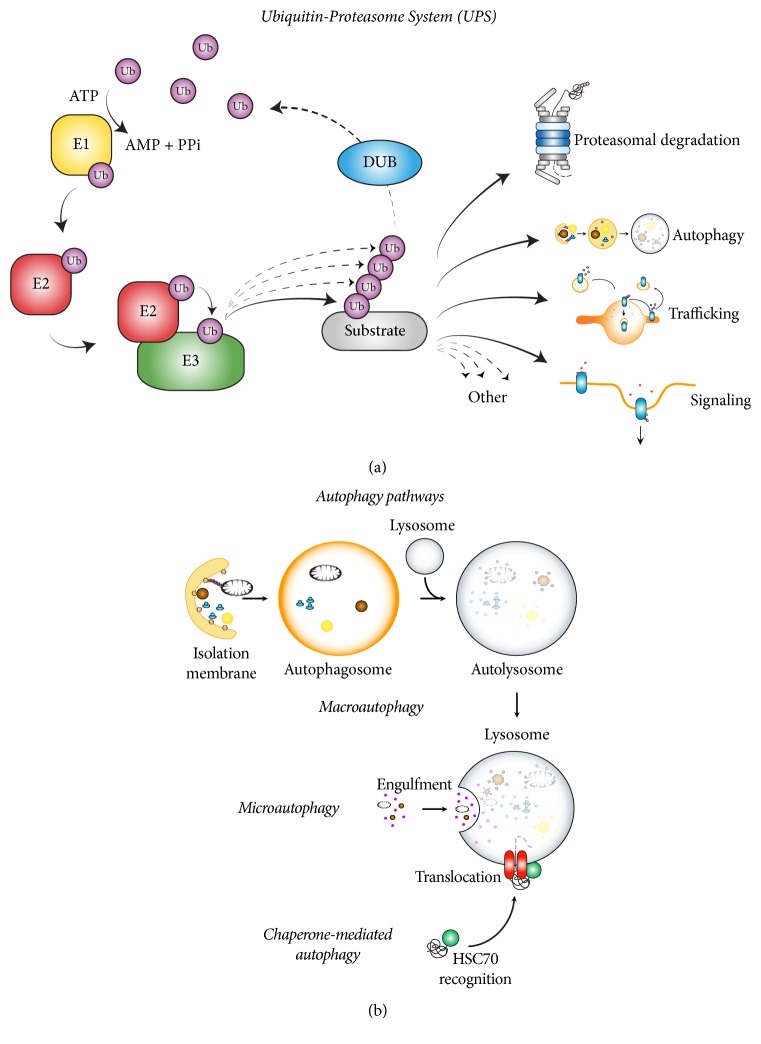
*Main intracellular quality control mechanisms: Ubiquitin-Proteasome System (UPS) and Autophagy. *(a) Ubiquitin is attached to target substrates by a sequential enzymatic cascade comprised by E1 (ubiquitin-activating), E2 (ubiquitin-conjugating), and E3 (ubiquitin-ligase) enzymes. E1 hydrolyses ATP to form an Ub-adenyl intermediate that is subsequently attached to the E1 via a thioester bond. E1-Ub transfers the ubiquitin to the E2, which then interacts with an E3 to transfer the ubiquitin to the substrate. DUBs can cleave ubiquitin moieties to edit ubiquitinated substrates. Protein ubiquitination regulates many biological processes, such as proteasomal degradation, autophagy, endosomal trafficking, and signalling events, and also chromatin assembly, DNA transcription and repair, ribosome biogenesis and translation, cell cycle and division, apoptosis, immunity, and organelle biogenesis. (b) Based on cargo recognition mechanisms, autophagy can be subdivided into macroautophagy, microautophagy, and chaperone-mediated autophagy. Macroautophagy is the best-studied form of autophagy, in which a double-membrane structure expands around and engulfs large cytosolic contents or organelles, forming an autophagosome. The autophagosome then fuses with a lysosome and the contents are degraded. Microautophagy degrades smaller cytosolic cargo, such proteins and tiny pieces of organelles by lysosomal invagination. CMA is involved in the degradation of unfolded or aggregated proteins that expose a particular degradation motif (KFERQ) that is then recognised by the cytosolic chaperone heat shock cognate protein of 70 kDa (HSC70), which interacts with lysosome-associated membrane protein type 2A leading to the unfolding and translocation of the substrate into the lysosomal lumen where it is degraded. Several macroautophagy subtypes can be distinguished according to cargo: reticulophagy (ER), mitophagy (mitochondria), pexophagy (peroxisome), ribophagy (ribosome), lipophagy (lipid droplets), xenophagy (intracellular pathogens such as bacteria and virus), and aggrephagy (protein aggregates).

Ubiquitin (Ub) is a small protein (~8.5 kDa) that is specifically attached to target proteins through a sequential enzymatic cascade [7]. Classically, Ub-activating E1 enzymes activate and transfer Ub to Ub-carrier E2 enzymes, which finally covalently modify the target proteins with Ub with the assistance of Ub-ligase E3 enzymes (Figure 1(a)). As is the case with other posttranslational modifications (PTMs), such as phosphorylation, ubiquitination is a reversible process. A fourth family of proteins, called deubiquitinases (DUBs), has the ability to cleave Ub moieties from their substrate proteins, acting as editors and recycling the free Ub pool. Conjugation of a single ubiquitin can be performed to a certain lysine of the target protein (monoubiquitination), or to several lysines simultaneously (multimonoubiquitination). Additionally ubiquitin can also be attached to another preassembled ubiquitin through the N-terminal, or any of its seven internal lysines, building chains (polyubiquitination) of different topology. Depending on which residue of the next ubiquitin is modified, M1, K6, K11, K27, K29, K33, K48, or K63 polyubiquitin chains can be formed. Combinations of alternate lysine residues can result in mixed ubiquitin chains too. Additionally, chains can be branched by other ubiquitin chains. Taken together, all these possible modifications result in a highly diverse set of chain types and ubiquitination types, each of which will have a different readout by the cell, the so-called “ubiquitin code” [9]. Due to this versatility of ubiquitin, the complexity of the UPS is extremely high and is not limited to play a role in protein degradation. Instead, UPS is essential in a plethora of additional key biological processes (Figure 1(a)), including receptor endocytosis and endosomal trafficking [10], cellular progression and chromosome reassembly, transcriptional regulation, signal transduction, and apoptosis [9].

Autophagy refers to the process in which cells engulf their own contents into double-membrane structures (autophagosomes) that ultimately fuse with lysosomes, where cargo is degraded and basic biomolecules are recycled back to the cytosol (Figure 1(b)) [8]. Large cytosolic contents or organelles are typically wrapped into a double membrane (isolation membrane) that expands engulfing cargo into autophagosomes (macroautophagy) [11]. Smaller cytosolic cargo is instead taken up by direct lysosomal invagination (microautophagy) [12], whereas unfolded or aggregated proteins are translocated into the lysosomal lumen by chaperone-mediated autophagy [13]. Interestingly, ubiquitination is also involved in the regulation of autophagy [14–19]. In addition to its other roles, therefore, it is clear that ubiquitination serves as universal tag for substrate degradation, as all intracellular degradation pathways appear to be interconnected and governed by it [20].

## 3. The UPS Is Essential for Correct Neuronal Homeostasis

Neurons particularly require a tight spatiotemporal regulation of their proteome. The cell body or soma is typically distant from axonal and synaptic connections; and they are constantly receiving, decoding, and transmitting information via synaptic communication. Regulation of protein interaction, sorting, and activity is not only critical for the wellbeing of the neuron itself, but it is also necessary for proper coordinated transfer of the information. Thus, right balance between protein synthesis and degradation is essential for neuronal homeostasis, both for correct neurodevelopment and, at later stages in aged neurons, to protect against stochastic proteotoxicity [21].

The first evidence of the involvement of the UPS in the nervous system homeostasis came from the discovery that ubiquitin is present in neurofibrillary tangles of various neurodegenerative diseases [22, 23]. Hereafter, a variety of failures at different levels of the UPS have been linked to several neurodevelopmental and neurodegenerative diseases. For instance, mutations in the UBA1 activating E1 enzyme are associated with X-linked Infantile Spinal Muscular Atrophy [24], whereas UBE2K E2 enzyme has been implicated in the pathogenesis of Huntington's disease [25, 26] and Alzheimer's disease [27]. UBE2H enzyme is associated with autism [28] and loss of Parkin and UBE3A ligase activity is linked to autosomal recessive juvenile parkinsonism and Angelman Syndrome, respectively [29, 30]. Similarly, downregulation of the DUB enzyme UCHL1 has also been linked with Parkinson's and Alzheimer's disease [31, 32]. Additionally, variants of the Ubiquilin1 (UBQLN1) ubiquitin receptor protein are associated with a higher risk of developing Alzheimer's disease [33], whereas disruption of the Rpt2 subunit of the proteasome in mice has been reported to be enough to trigger PD-like neurodegeneration [34]. Ubiquitin-mediated degradation and signalling are of outstanding importance for adequate neuronal function and development. Ubiquitination regulates processes such as neurite growth and guidance [35], synaptic maturation and neurotransmitter release [36, 37], and neurotransmitter receptor internalisation [38] and it is even imperative for neurogenesis to successfully take place [39].

*Drosophila* has been a valuable tool to shed light on our understanding of the role of ubiquitination in the nervous system. In fact, evidence of a link between UPS and synapse formation has often come first from experiments performed in flies. For example, in the early 90s, the* fat facets (faf)* gene was found to encode a DUB involved in fly eye development [40, 41], while the E2 enzyme coding* bendless* gene was shown to regulate neuronal connectivity [42, 43]. Fly mutants of the E3 ligase gene* highwire* were later reported to have a defective synaptic overgrowth and function in larval neuromuscular junction (NMJ) [44]. Similarly, another E3 ligase, the Anaphase Promoting Complex/Cyclosome, was shown to regulate synaptic size and synaptic transmission at fly NMJ [45]. Over the years, many other* Drosophila* studies have reported evidence of the involvement of the UPS in the nervous system development and function [46–50].

## 4. Studying UBE3A Function and Angelman Syndrome (AS) Employing* Drosophila*

The broad use of* Drosophila* as a model organism since the early years of the 20th century can be explained by its many advantages. First of all they are suitable for genetic studies as their fast reproductive cycle coupled to a great capacity to provide a large amount of eggs guarantees abundant offspring in short periods of time [51, 52]. They are easy and cheap to handle and maintain, which makes large-scale experiments affordable. Moreover, they only contain 4 pairs of chromosomes: the X/Y pair of sexual genes and three pairs of autosomal chromosomes [53], which greatly facilitates the management and interpretation of genetic experiments. In addition, the low genetic complexity of flies implies that there is less redundancy and simplifies biological and mechanistic explanations. Nevertheless, flies contain homologues for ~75% of human genes involved in disease [54], providing a simpler* in vivo *model for the study of their role in the context of many diseases, including neurodevelopmental and neurodegenerative diseases [55, 56].

**Figure 2:**
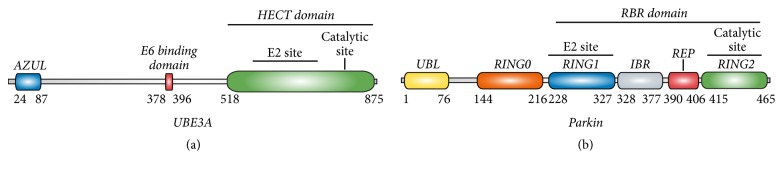
*E3 ligase types, UBE3A and Parkin.* (a) Human UBE3A domain structure. Protein domain structure and amino acid numbering refer to the isoform II. UBE3A contains an AZUL (amino-terminal Zn-finger of UBE3A E3 ligase) domain, thought to play a role in substrate recognition, as well as a HECT domain (Homologue to E6AP Carboxyl Terminus) characteristic of this family of E3 ligases, which was named after its discovery in UBE3A, also known by the name E6AP. The ubiquitin ligating catalytic cysteine is found within this HECT domain. All through the rest of the sequence of UBE3A only a small region known to interact with viral protein E6 has been described. (b) Parkin domain structure. Parkin contains a N-terminal UBL domain followed by a RING-like domain (RING0) and a RBR domain. The RBR domain entails a RING1 domain, which comprises the E2 binding site, a IBR domain, and the catalytic site encompassing RING2 domain. Amino acid numbering is based on human sequences.

Angelman Syndrome (AS) is a rare neurodevelopmental disorder with a prevalence of approximately 1/15.000 individuals [57], characterised by a severe intellectual and developmental delay, movement or balance disorders, speech impairment, and a happy demeanour that includes episodes of frequent laughter and easy excitability [58]. Very frequently (>80% of the cases) these symptoms are accompanied by seizures, sleep disturbances, and microcephaly [58, 59]. The underlying molecular cause leading to AS was discovered to be the loss of function of the UBE3A protein in the brain. In particular, mutations leading to truncated forms of UBE3A were found to be enough to develop the syndrome [29, 60]. UBE3A is a HECT-type ubiquitin E3 ligase enzyme (Figure 2(a)) of approximately 100 kDa [61], which according to* in vitro* studies catalyses attachment of K48-linked ubiquitin chains to its substrates, consequently targeting them for proteasomal degradation [62]. Interestingly, duplication of the* UBE3A* gene has been associated with autism [63–65]. Many attempts have been performed in order to identify the neuronal substrates of this enzyme, leading to the proposal of several candidate substrates. Some of the proposed substrates were only validated* in vitro* (Arc, Na+/K+ ATPase, p27, Ring1B, Adrm1, and Rpt5) and therefore cannot be concluded to be neuronal targets of UBE3A [66–70], while others were identified as ubiquitinated by UBE3A using nondenaturing immunoprecipitation approaches (Annexin A1, HHR23A, PSMD2, and Ephexin5), which means that the ubiquitin signal could well belong to any of the coprecipitating proteins [71–74]. Most importantly,* in vivo *validation of any of these candidates has been unsuccessful so far.

*Drosophila* UBE3A (Ube3a) is ubiquitously expressed during embryogenesis and is broadly detectable in the adult nervous system, particularly in the mushroom bodies, which represent the key region for learning and memory [75]. Different fly models have been generated to study AS and* UBE3A* duplication-based autism cases, reporting that* Ube3a* mutant flies mimic characteristics of human AS [75–78].* Ube3a* null mutant flies display locomotor impairment, abnormal circadian rhythms, and learning and memory defects, with a particular effect on long-term memory [75]. Furthermore, loss of Ube3a in neurons results in decreased dendritic arborisation of larval peripheral neurons [77] and decreased dopamine levels in adult fly brain [79]. In addition, neuronal overexpression of Ube3a also results in locomotion defects, in an ubiquitin-ligase-dependent manner. Missense mutations found in* UBE3A* alleles of AS patients have been reported to act as loss-of-function mutations also in its* Drosophila* homologue [75]. Fly models overexpressing Ube3a have been shown to display comparable neurotransmission defects to those found in mouse models of duplication 15q autism. Overexpression of wild-type Ube3a, but not its ligase-dead form, compromised the capacity of motor neuron axons to support closely spaced trains of action potentials, while at the same time increasing excitability [78]. Indeed, both overexpression and deficiency for Ube3a alter neurotransmission at the neuromuscular junction in* Drosophila melanogaster* 3rd instar larvae, also inducing in both cases defects in glutamatergic signalling [78]. A study investigating the role of Ube3a in the learning ability of flies using the aversive phototaxis suppression assay determined that both down- and upregulation of Ube3a are detrimental to learning in larvae and adults [80].

## 5. Parkin and Parkinson's Disease (PD), Lessons from* Drosophila*

Parkinson's Disease (PD) is the second most common neurodegenerative disease after Alzheimer's. It is considered to affect 1% of people older than 60 years and up to 4% older than 80 years [81, 82]. Parkinsonism englobes numerous neurological syndromes that are mainly characterised by resting tremor, rigidity, and postural disability. PD patients display these motor symptoms, usually accompanied by other nonmotor symptoms, including depression, constipation, hypotension, sleep disorders, and dementia. Pathologically, PD is mainly characterised by loss of dopaminergic neurons in the substantia nigra and the presence of Lewy bodies, intracytoplasmic proteinaceous inclusions rich in *α*-synuclein [83, 84]. However, the exact pathophysiological mechanisms leading to the disease are not clear yet and treatments modifying disease progression are not available. PD has been classically considered a sporadic disease linked to aging with an unknown aetiology. However, in about 10% of the cases, mutations in specific genes cause familial forms of PD [85]. Mutations in the RING-Between-RING (RBR) E3 ligase Parkin (Figure 2(b)) are the most frequent cause of all the autosomal recessive forms [86–89]. According to several structural studies, PD-causing mutations in Parkin result in loss of its function by either diminishing the E3 ligase activity or affecting the correct folding of the protein [87, 90–94].

Extensive studies performed employing* Drosophila *have been critical to improve our understanding of PD pathophysiology and Parkin function. In fact, the first hint that Parkin was involved in mitochondrial homeostasis came from the analysis of Parkin null flies (generated through ablation of endogenous* parkin* gene through P-element mutagenesis).* parkin* deficient flies display decreased dopamine content and dopaminergic neurodegeneration; they also reduced longevity, motor deficits, and male sterility [95–98]. Ultrastructural analyses showed that Parkin loss results in abnormally swollen and disorganised mitochondria, leading to apoptotic cell death of muscle tissue and defective spermatogenesis [95, 98]. Transcriptional analysis of* parkin* null flies revealed that mitochondrial electron transport chain genes, as well as genes involved in oxidative stress and innate immune responses, were upregulated [99]. In addition, the c-Jun N-terminal kinase pathway has been suggested to be upregulated in dopaminergic neurons of Parkin deficient flies, resulting in stress-mediated apoptotic neurodegeneration [96]. Ever since, Parkin has been acknowledged as a neuroprotective factor in many* in vitro *and* in vivo* studies [100] and, consequently, Parkin overexpression is associated with improved mitochondrial function, increased lifespan, and reduced proteotoxicity [101]. However, more recent studies in flies and cells are challenging this view, as Parkin overexpression can also have deleterious effects [102–104]. Seminal studies demonstrated that another PD-associated gene, coding for the mitochondrial kinase PINK1 [105], acts in the same pathway upstream of Parkin.* Pink1* null flies display the same defective phenotypes as* parkin* null flies, and Parkin overexpression can rescue* Pink1* loss but not vice versa [106–108]. Subsequent* Drosophila *genetic studies showed that* Pink1* and* parkin* interact with the mitochondrial fission and fusion machinery to regulate mitochondrial dynamics [109–111].

Mammalian cell culture studies first established that PINK1 accumulates on depolarised or damaged mitochondria to recruit and activate latent overexpressed Parkin and dispose of dysfunctional mitochondria via mitophagy [112, 113]. Thereafter, Pink1/Parkin-dependent mitophagy has also been detected in* Drosophila* S2R+ cells [114] and* in vivo Drosophila* models have reinforced mammalian cellular discoveries. Functional studies revealed that Parkin is phosphorylated by Pink1 in* Drosophila* cells, leading to Parkin activation. Parkin phosphorylation status modifies phenotypes typically affected in* Pink1* and* parkin* null mutant flies [115]; and mitochondrially located phospho-Ub (p-Ub) rescued* Pink1 *null associated defects, supporting the requirement of both ubiquitin and Parkin phosphorylation for Parkin activation in the Pink1/Parkin pathway [116]. Nevertheless, it remains formally unproven that PINK1 and Parkin promote mitophagy* in vivo* and that defects in the disposal of dysfunctional mitochondria are involved in the progression of the PD.

Recent findings have identified additional PD-associated genes involved in Parkin-dependent mitophagy.* Fbxo7* genetically interacts with* parkin* in* Drosophila* and is involved in PINK1/Parkin-dependent mitophagy in mammalian cells [117]. In addition,* parkin* has been shown to further genetically interact with* LRRK2* and* Vps35* in flies [118, 119], although the functional implications are yet to be elucidated. Beyond mitophagy, Parkin deficiency has been related to additional dysfunctions [120]. Parkin has been reported to ubiquitinate a broad range of substrates, including several Lewy body components, by interacting with different E2s and catalysing various ubiquitination types, preferentially K6-linked polyubiquitin chains [89, 91, 92, 121–124]. However, most of these studies were performed* in vitro* upon overexpression of the putative substrate and/or Parkin. Although several unbiased quantitative mass spectrometry studies have reported altered protein levels in Parkin deficient* Drosophila* and mice [125–128],* in vivo* Parkin substrates have not been identified so far.

## 6. Studying Ubiquitin Proteomics with Drosophila

Primary discoveries are usually performed* in vitro* or* in cellulo*, but successive* in vivo* confirmation is required when translation towards human health is sought.* Drosophila* represents an ideal organism to study ubiquitin pathways* in vivo*. Ubiquitin is highly conserved across all eukaryotes [129],* Drosophila* Ub being 100% identical to the human protein. In humans Ub is encoded by four genes:* UBA52*,* RPS27A (UBA80)*,* UBB*, and* UBC* [130, 131], while in* Drosophila* three homologous genes exist:* RpL40 (DUb52)*,* RpS27A (DUb80)*, and* Ubi-p63E* [132].

The* Drosophila* proteome is predicted to contain ~15.000 gene products, of which ~10.000 proteins have been successfully identified employing mass spectrometry (MS) [133, 134]. Studying ubiquitination* in vivo*, however, can be very challenging, particularly in neurons. Due to the low stoichiometry at which ubiquitin-modified proteins are present within cells, it is necessary to enrich the ubiquitinated fraction prior to the MS analysis [135]. For this purpose, several purification methods have been developed so far [66, 136–140]. Nevertheless, most of these enrichment methods require the purification to be performed under native conditions, copurifying contaminants and false positives [141]. Alternatively, ubiquitinated peptides rather than intact ubiquitinated proteins can be enriched prior to the MS analysis. Proteolytic digestion of the sample with trypsin produces a characteristic di-Gly signature in ubiquitinated peptides that is detectable by MS [136]. Specific antibodies that recognised this ubiquitin remnant have been developed in recent years [142] and used for the isolation and subsequent MS-based identification of thousands of putative ubiquitination sites* in vivo *[143, 144]. This approach, however, requires the proteins to be trypsinized preventing any immunoblotting on the purified material. Since other ubiquitin-like proteins, as well as certain experimental conditions, also leave this di-Gly signature in the peptides [145, 146], such orthogonal validations are essential.

To avoid the detection of false positive ubiquitinated proteins, an enrichment process under denaturing conditions is preferred over the usage of physiological buffers. This has been classically performed using poly-histidine tagging [136, 147, 148]. However, a relatively high number of endogenous histidine-rich proteins are found in higher eukaryotes, which are also trapped in the nickel affinity beads, resulting in excessive background. In order to overcome these limitations, we developed the ^bio^Ub strategy [149], based on a chemical modification performed by biotin holoenzyme synthetase enzymes [150] during the metabolism of fatty acids, amino acids, and carbohydrates [151]. This biotinylation reaction is highly specific and only few proteins are found to be modified with biotin* in vivo* [152]. The minimal length peptide that can be efficiently biotinylated by the* E. coli* biotin holoenzyme synthetase BirA is 14 amino acids long [153]. This can be used as a powerful tool for the generation of fusion proteins that can be easily purified or detected thanks to their biotin tag. The strategy for the* in vivo* isolation of ubiquitin conjugates has so far allowed the purification and enrichment of large amounts of ubiquitin conjugates from flies [104, 149, 154], mice [155], and human cell lines [156].

**Figure 3:**
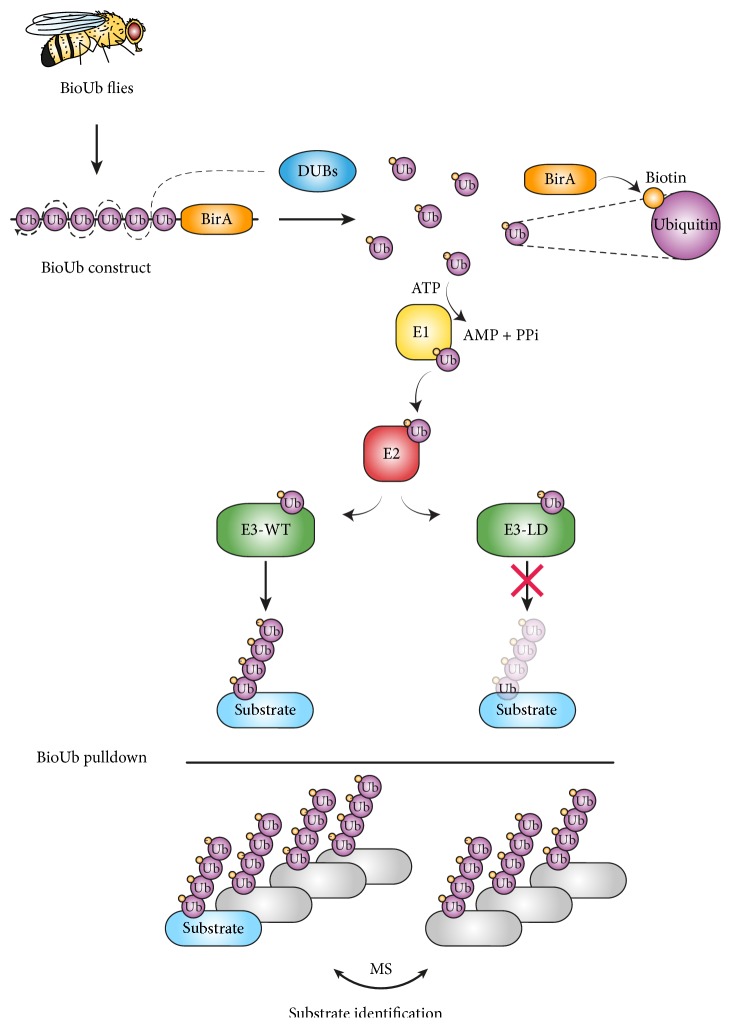
*BioUb strategy to identify ubiquitin substrates of E3 ligases in Drosophila neurons.* Scheme of the strategy used to identify proteins ubiquitinated by Parkin and Ube3a in* Drosophila* neurons. Flies were engineered to express endogenously precursor capable of biotinylating ubiquitin in* Drosophila *photoreceptors using the* GMR-GAL4* driver. This biotin modified ubiquitin (^bio^Ub) is then incorporated within the pool of endogenous ubiquitin, in flies that also overexpress wild-type E3 ligases (E3-WT), Parkin or Ube3a, and in their respective mutant or ligase-dead negative controls (E3-LD). Ubiquitinated material can then be purified using Neutravidin beads and isolated material analyzed by mass spectrometry (MS). Ubiquitinated proteins enriched in Parkin or Ube3a WT overexpressing neurons can then be identified based on both protein LFQ levels and peptide intensities.

The ^bio^Ub system relies on the* in vivo* expression of the ^bio^Ub construct, which is formed by six ubiquitin-coding sequences in tandem followed by the bacterial bifunctional ligase/repressor BirA enzyme (Figure 3). Endogenous DUBs digest the ^bio^Ub construct releasing BirA and ubiquitin and mirroring the processing of endogenous ubiquitin gene products [157]. Each ubiquitin contains a 16-amino-acid long biotinylatable motif, which is then recognised and biotinylated by BirA endogenously, resulting in a biotin-tagged ubiquitin moiety (^bio^Ub) that is efficiently handled by the cascade of ubiquitin-conjugating enzymes and successfully attached to target proteins together with the endogenous ubiquitin. The advantage of having ubiquitinated proteins tagged with biotin is that they can be very efficiently and specifically purified employing avidin-conjugated beads. Biotin-avidin interaction is one of the strongest identified interactions in nature [158, 159], and it allows carrying out the enrichment and washes of ubiquitinated material under very harsh conditions, such as 8 M Urea, 1 M NaCl, and 2% SDS, avoiding coisolation of nonubiquitinated interacting partners [149]. Finally, the isolated material can be subjected to MS or Western blot analysis [104, 149, 154–156].

**Figure 4:**
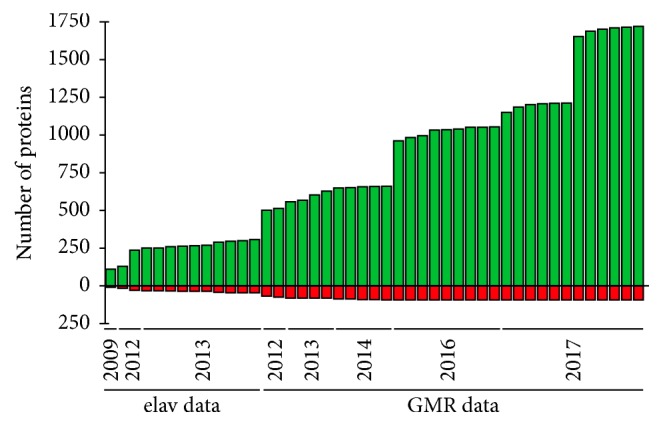
*Proteomic analysis of Drosophila neuronal ubiquitome.* A cumulative number of identified ubiquitinated proteins (green) isolated from* Drosophila* neurons by means of the ^bio^Ub approach are shown. A cumulative number of proteins that appear in control birA pulldowns, and which are therefore classified as background, are shown in red. The first analyses (until 2013) were obtained from fly embryonic developing neurons (using elav-GAL4 driver). Subsequent analyses were performed with proteins isolated from the* Drosophila* photoreceptor cells (employing GMR-GAL4 driver).

On our first application of this method, our group detected 121 ubiquitinated proteins in* Drosophila* neurons during embryonic development [149], including several key proteins involved in synaptogenesis and hence suggesting that UPS is important for proper neuronal arrangement. We later compared the ubiquitin landscape between developing and mature neurons in* Drosophila melanogaster* and identified 234 and 369 ubiquitinated proteins, respectively [154], some of which were found in both developmental stages. More interestingly, certain proteins are preferentially ubiquitinated in specific cell types during specific periods of the* Drosophila* life cycle, reinforcing the importance of using the appropriate cell type when studying ubiquitination. For example, Ube3a was found to be active in both developing and adult neurons, while Parkin was found to be enzymatically active in adult neurons only [104, 154]. Recently we have successfully employed this approach to analyze the ubiquitinated proteome of* Drosophila* under different conditions ([104, 154] and Ramirez et al. unpublished data). Altogether and thanks to the usage of more sensitive MS instruments, we have identified a total of ~1700 ubiquitinated proteins in* Drosophila* neurons (Figure 4), which represent ~11% of the total fly proteome (15.000).

## 7. Label-Free Quantitative Proteomics to Identify E3 Ligase's Ubiquitin Substrates

The ^bio^Ub strategy can be applied to identify ubiquitin substrates of selected E3 ligases by comparing the levels of ubiquitinated proteins in an E3 ligase-dependent manner (Figure 3). In fact, we have recently been pioneers in deciphering the ubiquitome of flies expressing the biotin-tagged ubiquitin in the context of either gain or loss of function of Parkin [104] and Ube3a (Ramirez et al., 2018* manuscript under review*) in adult Drosophila neuron. In both cases, to detect the E3-ligase substrates, we followed a label-free quantitative proteomics approach. Ubiquitinated proteins that were enriched using the ^bio^Ub strategy in each of the experimental conditions were independently analyzed by MS. Resulting MS raw files were subsequently combined for the bioinformatic analysis in which a search engine determined the identity of the proteins in the samples as well as their relative abundance. Consequently, those proteins, which were found to be more abundant in the presence of the wild-type version of the E3 ligase rather than in the presence of the ligase-dead version of the ligase, were considered putative E3 ligase substrates.

We successfully isolated >1.000 ubiquitinated proteins, identifying, for example, 37 proteins whose ubiquitination is affected by Parkin activity: 35 were more and 2 were less ubiquitinated [104]. These include proteins associated with the endosomal sorting complexes required for transport (ESCRT) machinery (ALiX, Vps4), subunits of the v-ATPase required for endosome and lysosomal acidification, and most importantly the PD-associated retromer component, Vps35. We validated several of these substrates, when* Drosophila* antibodies were available and interestingly showed that most of them were monoubiquitinated by Parkin. Furthermore, in agreement with previous mammalian cellular studies [124], ubiquitin chain-linkage analysis confirmed that Parkin preferentially catalyses K6-Ub chains* in vivo*.

In the case of Ube3a flies, several UPS and autophagy-related proteins were identified to be more ubiquitinated upon Ube3a overexpression, including two proteasomal interacting proteins (Rpn10 and Uch-L5) earlier identified by our lab as Ube3a substrates [160]. Our proteomic data in neuronal tissue corroborate the findings in mammalian cell culture that were earlier reported [70, 73]. That is, UBE3A regulates several proteasomal subunits, which makes it likely that further changes on the Ube3a-altered ubiquitome might be a secondary effect. In any case, several proteins with important roles in neuronal morphogenesis and synaptic transmission have also been detected.

In addition, to detect E3 ligase substrates, our investigation allowed us to gather information about specific ubiquitination sites as well as types of ubiquitination linkages. In most proteomic studies, trypsin is the enzyme of choice to digest proteins and obtain suitable peptides that are further analyzed by MS. When the conjugated ubiquitin is cleaved with trypsin, it leaves a Gly-Gly dipeptide remnant on the conjugated lysine residues that serve as a signature of ubiquitination and allows depicting the specific site of modification. In agreement with* in vitro* studies showing that UBE3A catalyses preferentially the attachment of K48-linked polyubiquitin chains [62, 161], we also observed in* Drosophila* that Ube3a induces K48 and K11 chains on its substrates. Interestingly, not all the validated substrates of Ube3a seem to be targeted for degradation [160] as one would have expected from these ubiquitin chain types.

## 8. Does Parkin Regulate Something More Than Mitochondrial Homeostasis?

Despite the fact that we identified some outer mitochondrial membrane proteins that have been reported to be ubiquitinated by Parkin during mitophagy, such as VDAC1/2/3, TOM70, and CISD1/2, mitochondrial proteins were not particularly enriched compared to previous studies [162, 163]. The restricted overlap between our dataset and other previous studies indicated that results from artificial cell culture conditions correlate with the biology of the brain within an organism only to a certain degree. Only 8 out of the 35 Parkin substrates identified by us have been identified in previous studies using mitochondrial depolarisation and mitophagy induction. In contrast, we captured the steady state substrates of Parkin* in vivo*, which might be involved in pathways beyond mitophagy. Our proteomic analysis of Parkin substrates revealed that Parkin ubiquitinates a wide range of proteins with no obvious functional connectivity, although endocytic trafficking components, such as Vps35, Vps4, or PDCD6IP/ALiX, were overrepresented. Interestingly,* parkin* has been recently shown to genetically interact with* Vps35* in* Drosophila* [119], and several studies have suggested that Parkin may be involved in endosomal trafficking [164, 165]. Additional studies will in fact reveal whether these substrates are functionally connected in a yet unknown pathway. Moreover, several proteins involved in transport of molecules and proteins; biosynthesis of proteins, carbohydrates, and lipids; ER stress; immunity and apoptosis were also identified in this large-scale ubiquitome study. The heterogeneity in the nature of the putative Parkin substrates detected suggests that the role of Parkin might be much wider than it is actually believed.

It is important to note that our* Drosophila* results, in contrast to previous studies, have not required promotion of Pink1 activity, and therefore we might have identified some Parkin substrates that are Pink1-independent. This opens the question of how Parkin can be activated then. It could be possible that the cleaved cytosolic Pink1 fragment may have a role in the activation of Parkin for other purposes than mitophagy. It can neither be discarded that other kinases have the ability to activate Parkin. Further studies depicting the requirement of Pink1 for the activation and ubiquitination of Parkin will clarify these questions.

## 9. Is UBE3A a Master Regulator of the Proteasome?

The* in vivo *unbiased proteomics approach we have performed has provided for the first time a list of putative Ube3a substrates, whose ubiquitination is enhanced by Ube3a. Additionally, our findings corroborate previous reports performed in cells, indicating that Ube3a interacts with the proteasome and its degradative activity, which results in the accumulation of tens of ubiquitinated proteins of which many are most likely not direct targets of Ube3a. The ubiquitination of proteasomal subunits by UBE3A had been previously reported, but this* Drosophila *study is pioneer in reporting* in vivo *evidence of their ubiquitination in neuronal cells. Complementing previous observations, it appears that the ubiquitination of proteasomal subunits by Ube3a/UBE3A ([70, 73, 160, 166]; Ramirez et al., 2018,* manuscript under review*) places this E3 ligase as a pivotal regulator of the proteasome and proteostasis. This finding opens a new perspective in which the ubiquitination of other proteins, and thus their levels or activity, can be affected as a downstream effect. The existing working model that UBE3A substrates are targeted for degradation does therefore need to be revised.

## 10. Concluding Remarks

The ^bio^Ub approach has been successfully applied for the MS analysis of the ubiquitin landscapes of the embryonic nervous system and* Drosophila* photoreceptor cells, but it has the potential to be implemented to any fly tissue at any stage during the development. The nature of the ^bio^Ub strategy allows also discerning by Western blot whether such identifications correspond to proteins that are mono- or polyubiquitinated* in vivo*. And most importantly, for the first time it is possible to obtain a list of candidate substrates for any* Drosophila* E3 ligase* in vivo*.
